# Effects of L-citrulline supplementation in the basal diet on reproductive performance, serum metabolites, and microbial community structure in Simmental cows

**DOI:** 10.3389/fmicb.2026.1742321

**Published:** 2026-03-03

**Authors:** Changgeng Li, Hui Chen, Jiaqi Liu, Weijie Zao, Chen Fan, Waike Lv, Guozhu Xu, Zhantao Lu, Xihu Wang, Xiaojun Liu, Guodong Zhao

**Affiliations:** 1 College of Animal Science, Xinjiang Agricultural University, Urumqi, China; 2 Xinjiang Agricultural Net Animal Husbandry Co., Ltd, Bole, China; 3 Boertala Mongolian Autonomous Prefecture Animal Disease Control and Diagnosis Center, Bole, China; 4 Hutubi Cattle Breeding Farm Co., Ltd., Hutubi, China

**Keywords:** L-Citrulline (L-Cit), reproductive hormones, ruminal microbiota, Simmental cows, untargeted metabolism

## Abstract

The experiment was designed to evaluate the influence of dietary supplementation with L-Citrulline (L-Cit) on reproductive outcomes, serum reproductive hormone profiles, ruminal and intestinal microbial communities, serum metabolites, and their metabolic associations in Simmental cows, thereby providing a theoretical basis for its application in ruminant production. All cows were screened by vaginal mucus microscopy (no pathogenic bacteria detected) and B-ultrasound examination (no ovarian cysts or uterine inflammation observed) to confirm the absence of reproductive disorders. A total of 240 multiparous Simmental cows, 3–4 years of age with an average body weight of 470 ± 15 kg, were randomly allocated into three groups of 80 animals each: a control group, Group I, and Group II. The control group received only the basal diet, whereas Group I and Group II were provided with the basal diet supplemented with 7 g/d and 14 g/d of L-Cit per cow, respectively. Day 0 of the trial was defined by the first intramuscular injection of prostaglandin (PG, 0.4 mg per cow), after which L-Cit supplementation commenced at 72-h intervals. On day 10, a second PG injection (0.4 mg per cow) was administered, and supplementation was discontinued on day 11. Estrus expression was monitored, and pregnancy was assessed on day 35 post-insemination using B-mode ultrasonography. During the experimental period, six cows were randomly selected from each group. Blood samples were collected from the coccygeal vein prior to the morning feeding on days 1 and 11 using plain tubes (without anticoagulant). The samples were centrifuged at 720 × g (approximately 3,000 rpm for 15 min) to separate the serum. The obtained serum was stored at −20 °C for subsequent analysis of reproductive hormones. In addition, blood, rumen fluid, and fecal samples were obtained 10 h after mounting to support untargeted metabolomic profiling and microbial community analysis. Compared with the control group, the estrus rate in experimental Group I increased by 12.5% (*p <* 0.05). In experimental Group II, serum concentrations of gonadotropin-releasing hormone (GnRH) and follicle-stimulating hormone (FSH) measured 10 h after cows accepted mounting rose by 15.79% (*p <* 0.05) and 35.71% (*p <* 0.01), respectively, relative to the control group. Analysis of 16S rRNA sequences revealed significant differences in ruminal microbial taxa, including *Verrucomicrobiota, Lentisphaeria, Oligosphaeraceae, vadinBE97_g_norank*, *vadinBE97*, *Eubacterium_ruminantium_group*, and *Prevotellaceae_g_norank*. In the intestine, significant differences were observed in *Lachnospiraceae*, *Lachnospirales*, *Marvinbryantia*, *Desulfovibrionia*, *Desulfovibrionaceae*, and *Desulfobacterota*. KEGG enrichment analysis based on Liquid Chromatography-Mass Spectrometry (LC–MS) *data indicated upregulation* of the arginine biosynthesis pathway in the experimental groups, whereas metabolites associated with the tricarboxylic acid (TCA) cycle were notably elevated in experimental Group I. Within the experimental framework, dietary supplementation with L-Cit combined with the two-shot PG synchronization protocol reshaped the ruminal microbial community structure of Simmental cows, improved glucose metabolic efficiency and xenobiotic degradation capacity, and promoted the synthesis and release of reproductive hormones. Consequently, estrus and conception rates were enhanced.

## 1 Introduction

L-Cit, a non-essential amino acid first identified in watermelon in 1914 (Banerjee, 2014), belongs to the *α*-amino acid family. In mammals, it participates in the urea cycle by enabling the conversion of ammonia into urea for excretion. Following intestinal absorption, L-Cit is transported to the kidneys, where argininosuccinate synthase (ASS) and argininosuccinate lyase (ASL) mediate its conversion to arginine (Sultana et al., 2002). The resulting arginine sustains nitric oxide (NO) and polyamine synthesis through nitric oxide synthase (NOS) (Yin et al., 2024). Acting as a signaling mediator, NO regulates vascular smooth muscle relaxation, mitigates oxidative stress, and contributes to follicular development, embryo implantation, and reproductive hormone secretion (Rosales and Jj, 1997; McHugh and Cheek, 1998; Zhou et al., 2021). In addition, NO represents the primary effector of penile erection and sexual arousal, thus maintaining essential functions in the male reproductive system (Argiolas, 1994).

Since arginine was demonstrated to improve reproductive performance in pregnant sows (Wu et al., 2009), its application has expanded across reproductive studies. Nevertheless, extensive ruminal degradation limits its direct efficacy in ruminants (Gilbreath et al., 2021). As a metabolic precursor of arginine, L-Cit avoids ruminal breakdown and ensures a reliable supply of bioavailable arginine (Gilbreath et al., 2019). Despite its potential, the mechanisms governing L-Cit absorption and metabolism in the rumen and intestines of ruminants remain insufficiently characterized.

Previous research demonstrated that dietary supplementation with L-Cit enhanced ram semen quality, enriched intestinal microbiota, and elevated serum reproductive hormone levels together with antioxidant capacity (Fan et al., 2024). In ewes, provision of 10 g/d L-Cit increased serum reproductive hormones, improved antioxidant capacity, and raised both lambing and twinning rates. Administration of L-Cit via drinking water in male mice resulted in higher serum levels of catalase (CAT), glutathione peroxidase (GSH-Px), hydroxyl radical scavenging activity, and total antioxidant capacity (T-AOC) (Ma et al., 2023; Zhao et al., 2023). Collectively, the evidence supports a regulatory function of L-Cit in animal reproductive performance.

Despite these observations, the intestinal absorption and metabolic mechanisms of L-Cit remain poorly defined. The present study employed multiparous Simmental cows to investigate dose-dependent effects of L-Cit, with the objective of elucidating its role in reproductive regulation by examining host metabolic pathways and intestinal as well as ruminal microecology. The outcomes are expected to establish a theoretical foundation for applying L-Cit in nutritional strategies for ruminants.

## 2 Materials and methods

### 2.1 Experimental instruments and materials

#### 2.1.1 Experimental drugs

L-Cit, (purity ≥ 98%) was obtained from Hebei Hongtao Biological Engineering Co., Ltd. Cloprostenol Injection (PG, 0.2 mg per vial) was purchased from Ningbo No.2 Hormone Factory. Enzyme-linked immunosorbent assay (ELISA) kits for gonadotropin-releasing hormone (GnRH, catalog number: ml410381), follicle-stimulating hormone (FSH, catalog number: ml497803), luteinizing hormone (LH, catalog number: ml440758), and estradiol (E_2_, catalog number: ml450381) were sourced from Shanghai Enzyme-linked Biotechnology Co., Ltd.

#### 2.1.2 Experimental equipment

The equipment included a veterinary B-mode ultrasound scanner (Kaixin Brand, Model RKU 10, Xuzhou Kaixin Technology Co., Ltd.), a microplate reader (Model iMark, Bio-Rad Laboratories, Inc.), a centrifuge (Model 5,424, Eppendorf AG, Germany), insemination gun, vacuum blood collection tubes, electronic balance, disposable syringes, gloves, and long-arm disposable gloves.

### 2.2 Animal feeding and management

Throughout the trial period, all cows were maintained under standardized feeding conditions. The housing environment was controlled at a temperature of 15–30 °C and a relative humidity of 40–50%. Pens were cleaned and disinfected regularly every day to ensure a clean and hygienic environment. The basal diet was provided in two equal meals daily at 8:00 and 18:00. Residual feed was removed 30 min after each feeding, and feed intake was recorded. Clean tap water was available ad libitum throughout the experiment. The detailed nutritional composition and levels of the diet are presented in Table 1.

**Table 1:** Dietary composition and nutritional levels (on a dry matter basis) %.

Ingredient	Content	Ingredient	Content
TMR ingredients	–	Nutrient Level 2	–
Straw	20	Moisture	7.89
Ensiled corn	35	Crude protein (CP)	13.10
Yellow silage	25	Crude fat (EE)	2.67
Corn	13	Crude ash (CA)	8.6
Cottonseed meal	6	Acid detergent fiber (ADF)	16.62
Premix 1	1	Neutral detergent fiber (NDF)	30.55
Total	100	Total phosphorus (TP)	0.37
–	–	Sulfur (S)	0.14
–	–	Dry matter (DM)	88.11
–	–	Calcium (Ca)	0.78
–	–	Chlorine (Cl)	0.56
–	–	Potassium (K)	1.16
–	–	Magnesium (Mg)	0.22

(1) The premix provides the following per kilogram of concentrate supplement: vitamin A: 12,000 IU, vitamin D₃: 3,500 IU, vitamin E: 80 IU, copper: 15 mg, zinc: 60 mg, manganese: 40 mg, iodine: 0.5 mg, selenium: 0.3 mg, cobalt: 0.2 mg, niacin: 150 mg, biotin: 2 mg. (2) All nutritional levels are actual measured values.

Conventional nutrients and mineral elements were analyzed according to the corresponding Chinese National Standards (GB/T). The specific methods were as follows: dry matter (DM) was determined by oven-drying at 105 °C to constant weight (GB/T 6435-2014); crude protein (CP) by the fully automated Kjeldahl method (GB/T 6432-2018); ether extract (EE) by Soxhlet extraction using anhydrous ether as the solvent (GB/T 6433-2006); ash by ashing in a muffle furnace at 550 °C (GB/T 6438-2007); acid detergent fiber (ADF) and neutral detergent fiber (NDF) by the Van Soest method (GB/T 20806-2006); calcium (Ca) by potassium permanganate titration (GB/T 6436-2018); total phosphorus (Total P) by ammonium metavanadate molybdate spectrophotometry at 420 nm (GB/T 6437-2018); sulfur (S) by barium sulfate turbidimetry at 400 nm (GB/T 17767.3-2008); chlorine (Cl) by ammonium thiocyanate back titration (GB/T 6439-2007); potassium (K) and magnesium (Mg) by atomic absorption spectrometry (GB/T 13885-2017). All analytical procedures were performed with reference to the method described by Yue et al. (2025).

### 2.3 Experimental design

The trial was carried out at Xinjiang Nongwang Animal Husbandry Farm (Longitude: 81.606239, Latitude: 44.993801) between September 2024 and May 2025. A total of 240 Simmental cows, 3–4 years of age and averaging 470 ± 15 kg body weight, were enrolled and allocated into four cohorts in September 2024, October 2024, April 2025, and May 2025, with 60 cows per cohort. Each cohort was subdivided into three groups of 20 animals.

All cows received 0.4 mg prostaglandin (PG) by intramuscular injection, with the initial administration designated as Day 0 to synchronize physiological status through corpus luteum regression. L-Cit supplementation began on Day 3. The control group received only the basal diet, whereas Experimental Group I and Group II were provided the basal diet supplemented with 7 g/d and 14 g/d of L-Cit per cow, respectively. Twenty Simmental cows were housed per pen. L-citrulline was thoroughly mixed with bursting corn and then divided equally into 20 portions, which were delivered into the feeding trough to ensure equal supplementation for each animal corresponding to 0.035% or 0.07% (w/w). The supplemented diet was delivered at 16:00 daily in equal portions. Feeding was maintained until Day 10, when a second PG injection (0.4 mg per cow) was administered, after which L-Cit supplementation was discontinued on Day 11. Estrus detection was performed three times daily (06:00, 12:00, and 18:00) for 30 min each by two trained observers, totaling 1.5 h d^−1^. Estrus was confirmed when a cow stood to accept mounting. Artificial insemination was conducted 10 h after estrus onset. Pregnancy diagnosis was performed by B-mode ultrasonography on Day 35 post-insemination, and conception rates were subsequently determined.

### 2.4 Sample collection and processing

#### 2.4.1 Blood sample collection

On Days 1 and 11, prior to morning feeding, six cows from each group were randomly selected, and 10 mL of blood was collected from the caudal vein using plain blood collection tubes. To prevent metabolite degradation, the blood samples were centrifuged at 720 × g within 30 min of collection. The supernatant (serum) was then aliquoted into 5 mL cryopreservation tubes within 1 h after centrifugation, flash-frozen in liquid nitrogen, and stored for subsequent analysis.

#### 2.4.2 Rumen fluid and fecal sample collection

After Day 10, six cows from each group were randomly selected. At 10 h post-mounting, 10 mL of rumen fluid was collected by repeatedly inserting and repositioning a rumen cannula sampler at multiple sites within the rumen digesta. Concurrently, 10 g of fecal samples were obtained via rectal collection. All samples were immediately aliquoted into multiple 5 mL cryopreservation tubes, flash-frozen in liquid nitrogen, and subsequently transferred to a − 80 °C freezer for long-term storage pending microbiome analysis.

#### 2.4.3 Sample determination

Serum levels of GnRH, FSH, LH, and E_2_ were quantified using ELISA kits. Liquid chromatography-mass spectrometry (LC–MS) analysis of serum metabolites was performed by Beijing Huatai Boao Biological Gene Co., Ltd., following the protocol described by Klepacki et al. (2016). Sequencing of 16S rRNA in rumen fluid and fecal microbiota was also conducted by the same company. Polymerase chain reaction (PCR) amplification employed bacterial primers (338F: 5’-ACTCCTACGGGAGGCAGCAG-3′ and 806R: 5’-GGACTACHVGGGTWTCTAAT-3′), and the detection procedure followed the method of Klepacki et al. (2016).

### 2.5 Statistical methods

Data on estrus and conception rates were initially organized using Microsoft Excel 2022. Differences in reproductive parameters among groups were compared using the chi-square test in SPSS 27. Hormone indicators were analyzed by one-way analysis of variance (ANOVA), with Duncan’s multiple range test employed for intergroup comparisons. Rumen fluid samples and untargeted metabolomics data were analyzed via the cloud platform of Beijing Huatai Bio-AO Biological Gene Co., Ltd.

Correlations among reproductive hormones, rumen microbiota, and serum metabolites were analyzed using Origin software. The Pearson correlation coefficient (parametric) was applied, and data visualization was performed with GraphPad Prism 9.0 and Origin Pro 2024b. Results are presented as mean ± standard error (SE). Differences were considered statistically significant at *p* < 0.05 and highly significant at *p* < 0.01.

## 3 Results and analysis

### 3.1 Effects of dietary supplementary feeding of L-Citrulline on Estrus rate and conception rate of Simmental cows

**Figure 1:**
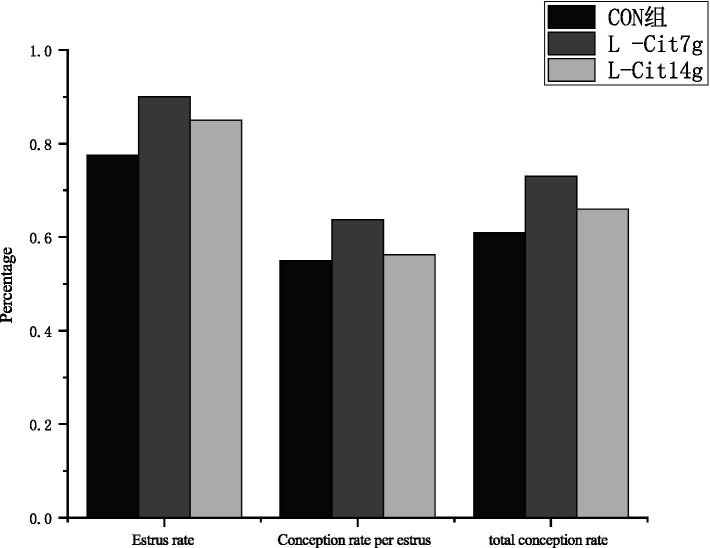
Effects of L-citrulline supplementation on estrus and conception rates in Simmental cows. The *x*-axis and *y*-axis represent the measured parameters and their respective proportions, respectively. Group I showed a significant increase in estrus rate compared to the control group.

In Figure 1, 80 Simmental cows were assigned to the control group, Experimental Group I, and Experimental Group II. Relative to the control group, the estrus rate increased by 12.5% in Experimental Group I (*p <* 0.05) and by 5% in Experimental Group II (*p >* 0.05). When compared with Experimental Group II, the estrus rate in Experimental Group I was 7.5% higher (*p >* 0.05). The estrous cycle conception rate of Experimental Group I exceeded that of the control group and Experimental Group II by 8.75% (*p >* 0.05) and 7.5% (*p >* 0.05), respectively, whereas Experimental Group II exhibited only a 1.25% increase compared with the control (*p >* 0.05). Similarly, the total conception rate in Experimental Group I surpassed that of the control group and Experimental Group II by 13.73% (*p >* 0.05) and 11.76% (*p >* 0.05), respectively, while Experimental Group II showed only a 2.22% increase relative to the control (*p >* 0.05).

### 3.2 Effects of dietary supplementary feeding of L-Citrulline on serum reproductive hormone levels of Simmental cows

**Figure 2:**
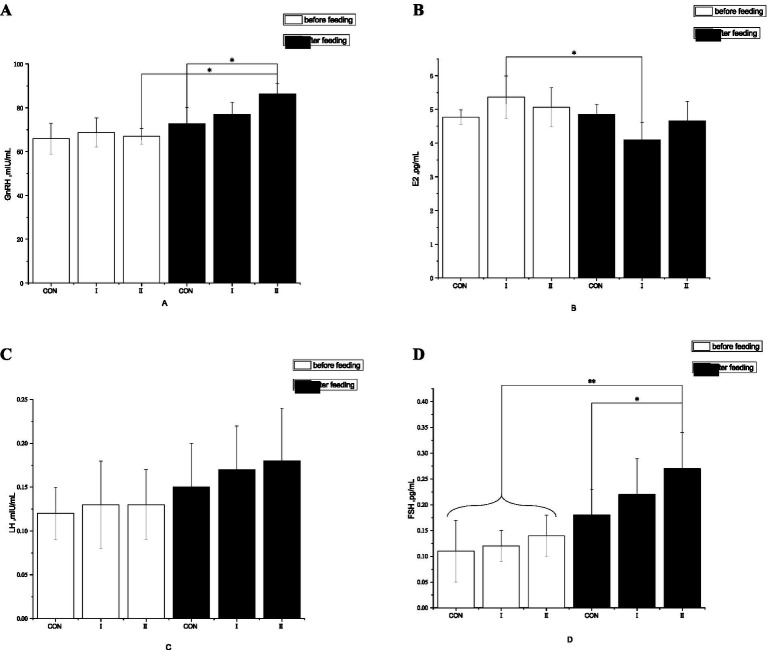
Effects of L-Citrulline supplementation on reproductive hormones in Simmental cows. The *x*-axis and *y*-axis represent the experimental groups and the concentrations of reproductive hormones, respectively. Group II exhibited a significant increase in serum GnRH levels compared to the control group. An asterisk denotes statistical significance: *p < 0.05. **(A)** represents the level of GnRH; **(B)** represents the level of E2 (estradiol); **(C)** represents the level of LH; **(D)** represents the level of FSH.

As presented in Figure 2, no significant intergroup differences were detected before *L-*Cit supplementation (*p >* 0.05). Following supplementation, serum GnRH levels rose by 5.47% in Experimental Group I (*p >* 0.05) and by 15.79% in Experimental Group II (*p <* 0.05) relative to the control group, with no significant variation between the two experimental groups (*p >* 0.05).

Regarding serum FSH levels, Experimental Group I and Experimental Group II exhibited increases of 21.74% (*p >* 0.05) and 35.71% (*p <* 0.05), respectively, compared with the control group, yet no significant difference was observed between the two experimental groups (*p >* 0.05).

### 3.3 Effects of dietary supplementary feeding of L-Citrulline on rumen microbiota of Simmental cows

#### 3.3.1 Alpha diversity analysis and beta diversity analysis

**Figure 3:**
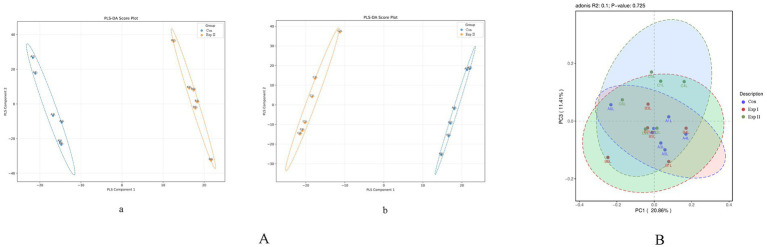
PLS-DA Score Plot and PCA plots. **(A-a)** Control group vs. Experimental Group I. **(A-b)** Control group vs. Experimental Group II. **(B)** Control group vs. Experimental Group I vs. Experimental Group II.

Table 2 shows that no significant variation was detected in any alpha diversity indices between the control and treatment groups, indicating that *L-*Cit supplementation did not alter ruminal microbial diversity. Sample coverage across all groups exceeded 99.9%, confirming that sequencing depth was adequate to capture the microbial composition of rumen fluid with high reliability. Furthermore, as depicted in Figure 3, the sample points from different groups largely overlapped, and their confidence ellipses intersected. A PERMANOVA (Adonis) test yielded a *p*-value greater than 0.05, indicating no significant difference in the overall microbial community structure among the three groups.

**Table 2:** Effects of dietary supplementation with L-Citrulline on rumen microbiota alpha diversity.

Item	Control group (CON)	Experimental Group I	Experimental Group II
Chao	2840.67 ± 114.37	2794.00 ± 92.85	2,773 ± 90.76
Ace	2837.83 ± 111.12	2800.67 ± 88.59	2775.83 ± 76.40
Shannon	6.44 ± 0.10	6.41 ± 0.06	6.35 ± 0.15
iCoverage	0.99 ± 0.00042	0.99 ± 0.00032	0.99 ± 0.0004
Simpson	0.005 ± 0.0007	0.005 ± 0.0007	0.007 ± 0.0041

#### 3.3.2 PLS-DA analysis of rumen microbiota

Figure 3 presents the PLS-DA (Partial Least Squares-Discriminant Analysis) score plots, which visually represent the classification efficiency of the model. In the comparison between the Control Group (CON) and Experimental Group I, the degree of separation between sample clusters reflected clear group discrimination, with the first and second principal components explaining 41.7 and 8.12% of the variance, respectively.

Similarly, in the comparison of CON with Experimental Group II, the sample distribution also demonstrated distinct segregation, with explained variances of 42.67% for the first principal component and 7.04% for the second.

#### 3.3.3 Effects of supplementary feeding of L-Citrulline on rumen microbiota of Simmental cows (phylum level)

**Figure 4:**
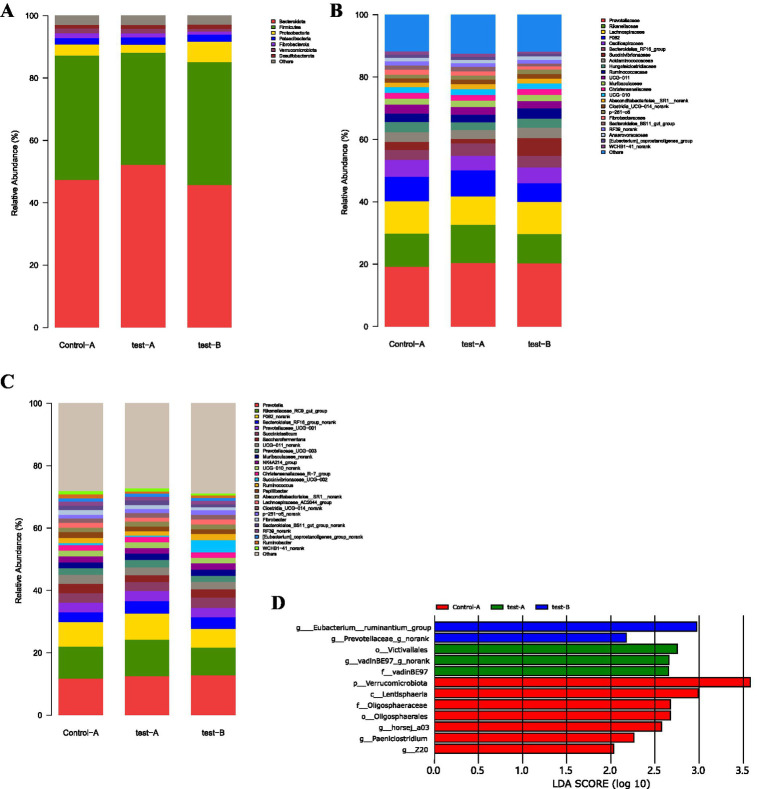
Relative abundance plots of rumen microbiota and linear discriminant analysis (LDA) bar plot. **(A)** Phylum level. **(B)** Family level. **(C)** Genus level. **(D)** Linear discriminant analysis (LDA) bar plot. For panels **(A–C)**, the *x*-axis and *y*-axis represent the experimental groups and the relative abundance (%) of microbiota at the phylum, family, and genus levels, respectively. For panel (D), the *x*-axis and *y*-axis represent the LDA score (log₁₀) and microbial taxa, respectively. The LDA bar plot statistically identifies microbial taxa with significant differential effects among groups. The LDA score, derived from linear discriminant analysis, indicates the effect size of each taxon’s abundance on group separation; a higher LDA score denotes a greater impact.

Figure 4A illustrates the phylum-level distribution of ruminal microbiota under L-Cit supplementation. The 10 most abundant phyla were *Bacteroidota*, *Firmicutes*, *Proteobacteria*, *Verrucomicrobiota*, *Patescibacteria*, *Fibrobacterota*, *Desulfobacterota*, *Spirochaetota*, *Cyanobacteria*, and *Actinobacteriota*. *Bacteroidota* and *Firmicutes* represented the predominant phyla across all groups.

Notably, *Bacteroidota* exhibited the highest relative abundance in Experimental Group II (52.16%), whereas *Firmicutes* were most abundant in the CON group (39.87%).

#### 3.3.4 Effects of supplementary feeding of L-Citrulline on rumen microbiota of Simmental cows (family level)

As shown in Figure 4B, analysis at the family level revealed that the 10 most abundant ruminal taxa included *Prevotellaceae*, *Rikenellaceae*, *Lachnospiraceae*, *F082*, *Oscillospiraceae*, *Bacteroidales_RF16_group*, *Succinivibrionaceae*, *Acidaminococcaceae*, *Hungateiclostridiaceae*, and *Ruminococcaceae*. *Prevotellaceae*, *Ruminococcaceae*, and *Lachnospiraceae* represented the dominant families in both the control (CON) and experimental groups, with higher relative abundances observed in Experimental Group I.

*Prevotellaceae* and *Ruminococcaceae* reached 20.38 and 12.25%, respectively. In contrast, *Firmicutes* exhibited a higher relative abundance in the CON, reaching 10.36%.

#### 3.3.5 Effects of supplementary feeding of L-Citrulline on rumen microbiota of Simmental cows (genus level)

At the genus level Figure 4C, the top 10 genera were *Prevotella*, *Rikenellaceae_RC9_gut_group*, *F082_norank*, *Bacteroidales_RF16_group_norank*, *Prevotellaceae_UCG-001*, *Succiniclasticum*, *Saccharofermentans*, *UCG-011_norank*, *Prevotellaceae_UCG-003*, and *Muribaculaceae_norank*. Among them, *Prevotella*, *Rikenellaceae_RC9_gut_group*, and *F082_norank* were particularly enriched in Experimental Group I, indicating their dominance within the ruminal microbiota.

*Prevotella* reached the highest relative abundance in Experimental Group I at 23.42%. *Ruminococcus* also displayed higher levels in the experimental groups compared with the CON, with relative abundances of 12.47% in Experimental Group I, 11.74% in Experimental Group II, and 8.37% in the CON.

#### 3.3.6 Differential species analysis

Figure 4D illustrates that 12 microbial taxa displayed significant differences between the control group and the experimental groups, comprising 1 phylum, 1 class, 2 orders, 2 families, and 6 genera. In the control group, 7 taxa were enriched, including *Verrucomicrobiota* (phylum), *Lentisphaeria* (class), *Oligosphaeraceae* (family), Oligosphaerales (order), *g_horsej_a03*, *Paeniclostridium* (genus), and *g_Z20*. Experimental Group I exhibited 3 taxa, namely Victivallales (order), *vadinBE97_g_norank*, and *vadinBE97*, whereas experimental Group II contained 2 taxa, *Eubacterium_ruminantium_group* and *Prevotellaceae_g_norank*. Effects of dietary supplementary feeding of L-Citrulline on intestinal microbiota of Simmental cows.

#### 3.3.7 Alpha diversity analysis and beta diversity analysis

**Figure 5:**
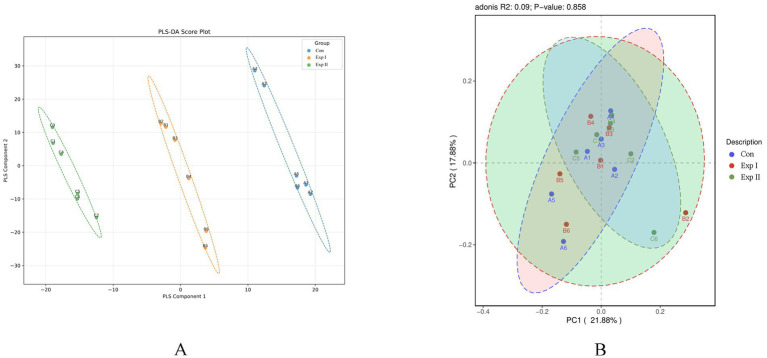
PLS-DA Score Plot and PCA plots. **(A)** Control group vs. Experimental Group I vs. Experimental Group II. **(B)** Control group vs. Experimental Group I vs. Experimental Group II.

Table 3 shows that no significant differences were observed in any alpha diversity indices of the intestinal microbiota between the control and treatment groups, indicating that L-Cit supplementation did not alter the overall intestinal microbial diversity. The species coverage in all groups exceeded 99.9%, confirming that the sequencing depth was sufficient to adequately capture the microbial composition of the fecal samples. As illustrated in Figure 5, sample points from different groups exhibited extensive overlap, and their confidence ellipses intersected. A PERMANOVA (Adonis) test yielded a *p*-value greater than 0.05, indicating no significant difference in the microbial community structure among the three groups.

**Table 3:** Effects of dietary supplementation with L-Citrulline on intestinal microbiota alpha diversity.

Item	Control group (CON)	Experimental Group I	Experimental Group II
Chao	1790.5 ± 134.7	1705.33 ± 109.20	1873.83 ± 119.37
Ace	1817.67 ± 127.51	1704.83 ± 106.83	1876.5 ± 127.71
Shannon	5.73 ± 0.1	5.67 ± 0.19	5.71 ± 0.18
Coverage	0.99 ± 0.0006	0.99 ± 0.0003	0.99 ± 0.0009
Simpson	0.0118 ± 0.002	0.0126 ± 0.003	0.0121 ± 0.002

#### 3.3.8 PLS-DA analysis of intestinal microbiota

Figure 5 presents the PLS-DA score plot, which visually represents the classification performance of the model. Greater separation among the three groups corresponds to more distinct classification. The first principal component accounted for 33.68% of the variance, while the second explained 5.23%.

### 3.4 Effects of dietary supplementary feeding of L-Citrulline on intestinal microbiota of Simmental cows

#### 3.4.1 Effects of supplementary feeding of L-Citrulline on intestinal microbiota of Simmental cows (phylum level)

**Figure 6:**
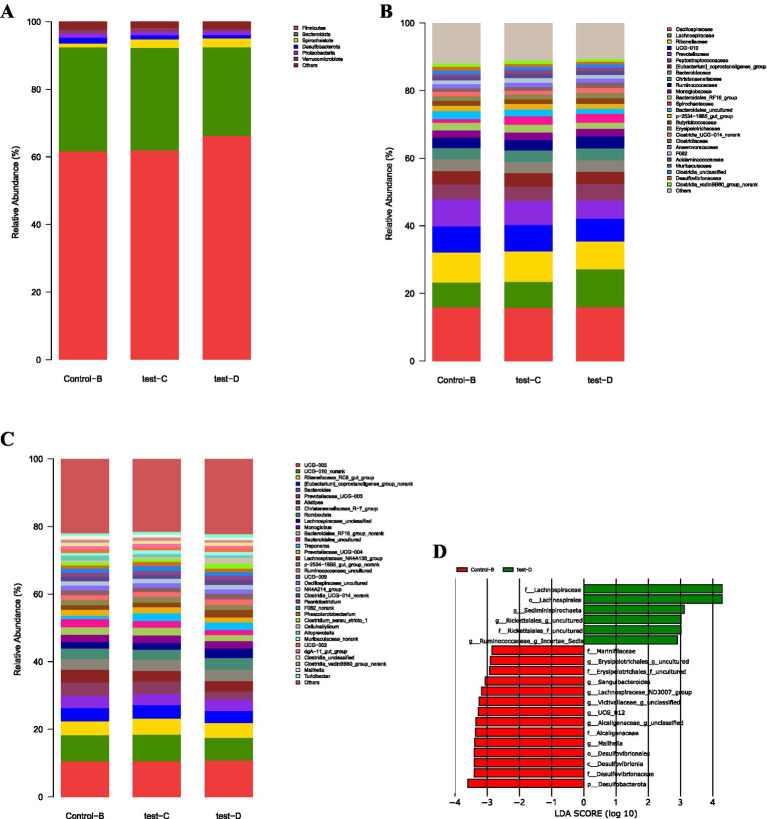
Relative abundance plots of intestinal microbiota and linear discriminant analysis (LDA) bar plot. **(A)** Phylum level. **(B)** Family level. **(C)** Genus level. **(D)** Linear discriminant analysis (LDA) bar plot. For panels **(A–C)**, the x-axis and y-axis represent the experimental groups and the relative abundance (%) of microbiota at the phylum, family, and genus levels, respectively. For panel (D), the x-axis and y-axis represent the LDA score (log₁₀) and microbial taxa, respectively. The LDA bar plot statistically identifies microbial taxa with significant differential effects among groups. The LDA score, derived from linear discriminant analysis, indicates the effect size of each taxon’s abundance on group separation; a higher LDA score denotes a greater impact.

As illustrated in Figure 6A, the dietary supplementation of *L-*Cit influenced the intestinal microbiota composition of Simmental cows at the phylum level. The eight phyla with the highest relative abundance were *Firmicutes*, *Bacteroidota*, *Spirochaetota*, *Proteobacteria*, *Actinobacteriota*, *Verrucomicrobiota*, and *Cyanobacteria* (note: although described as “top 8,” only seven phyla are listed, consistent with the original text).

*Firmicutes* and *Bacteroidota* exhibited consistently high proportions across the Control Group, Experimental Group I, and Experimental Group II, thereby representing the dominant phyla. In Experimental Group II, the relative abundance of *Firmicutes* reached 66.24%, exceeding the levels observed in the other groups. Conversely, *Bacteroidota* demonstrated the greatest relative abundance in the Control Group, reaching 30.77%, which was higher than that observed in the experimental groups.

#### 3.4.2 Effects of supplementary feeding of L-Citrulline on intestinal microbiota of Simmental cows (family level)

As shown in Figure 6B, the analysis of dietary *L-*Cit supplementation on the intestinal microbiota of Simmental Cows at the family level revealed that the top 10 families in relative abundance were *Oscillospiraceae*, *Lachnospiraceae*, *Rikenellaceae*, *UCG-010*, *Prevotellaceae*, *Peptostreptococcaceae*, *coprostanoligenes_group*, *Bacteroidaceae*, *Christensenellaceae*, and *Ruminococcaceae*.

*Oscillospiraceae*, *Lachnospiraceae*, and *Rikenellaceae* represented the major families across the Control Group, Experimental Group I, and Experimental Group II. *Oscillospiraceae* and *Lachnospiraceae* showed the highest proportions in Experimental Group II, with relative abundances of 15.92 and 12.21%, respectively. In contrast, *Rikenellaceae* displayed its highest relative abundance in Experimental Group I, accounting for 8.98%.

#### 3.4.3 Effects of supplementary feeding of L-Citrulline on intestinal microbiota of Simmental cows (genus level)

As illustrated in Figure 6C, analysis of the intestinal microbiota at the genus level following dietary supplementation with *L-*Cit in Simmental cows revealed that the 10 genera with the highest relative abundance were *UCG-005*, *UCG-010_norank*, *Rikenellaceae_RC9_gut_group*, *coprostanoligenes_group_norank*, *Bacteroides*, *Prevotellaceae_UCG-003*, *Alistipes*, *Christensenellaceae_R-7_group*, *Romboutsia*, and *Lachnospiraceae_unclassified*.

Among them, *UCG-005*, *UCG-010_norank*, and *Rikenellaceae_RC9_gut_group* displayed greater proportions across the Control Group, Experimental Group I, and Experimental Group II, thereby representing the dominant genera. In Experimental Group II, UCG-005 showed the highest relative abundance (10.74%), while *UCG-010_norank* (7.86%) and *Rikenellaceae_RC9_gut_group* (4.75%) were more prevalent in Experimental Group I compared with the other groups.

#### 3.4.4 Differential species analysis

As shown in Figure 6D, 20 microbial taxa with significant differences were detected between the Control Group and the experimental groups, including 1 phylum, 2 orders, 6 families, and 11 genera. Within the Control Group, 13 taxa were enriched, including *Marinifilaceae,* an unclassified genus of *Erysipelotrichales,*an unclassified family of *Erysipelotrichales*; V*ictivallaceae, Sanguibacteroides*, *Lachnospiraceae_ND3007_group*, an unclassified genus of *Victivallaceae*, *UCG_012*, an unclassified genus of *Alcaligenaceae*, *Alcaligenaceae*, *Mailhella*, *Desulfovibrionales*, *Desulfovibrionia*, *Desulfovibrionaceae*, and *Desulfobacterota*.

In Experimental Group I, 6 taxa were identified: *Lachnospiraceae, Lachnospirales, Sediminispirocheata, Marvinbryantia*, an uncultured genus of *Rickettsiales*, an uncultured family of *Rickettsiales,* and an uncultured genus of *Ruminococcaceae*a. Effects of dietary supplementary feeding of L-Citrulline on serum metabolites of Simmental cows

#### 3.4.5 PLS-DA analysis of serum

**Figure 7:**
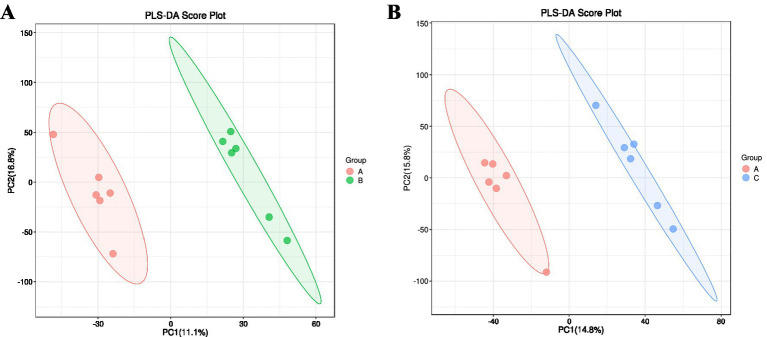
PLS-DA score plot. **(A)** Control group vs. experimental Group I. **(B)** Control group vs. experimental Group II. The *x*-axis and *y*-axis represent principal component 1 and principal component 2, respectively.

Figure 7 illustrates the PLS-DA (Partial Least Squares-Discriminant Analysis) score plots used to visualize model-based group discrimination. In the comparison between CON and Experimental Group I, clear separation was observed, indicating effective group differentiation; the first and second principal components accounted for 11.1 and 16.8% of the variance, respectively.

In the comparison between CON and Experimental Group II, the separation was similarly distinct, with the first and second principal components explaining 14.8 and 15.8% of the variance, respectively.

#### 3.4.6 Screening of differential serum metabolites

**Figure 8:**
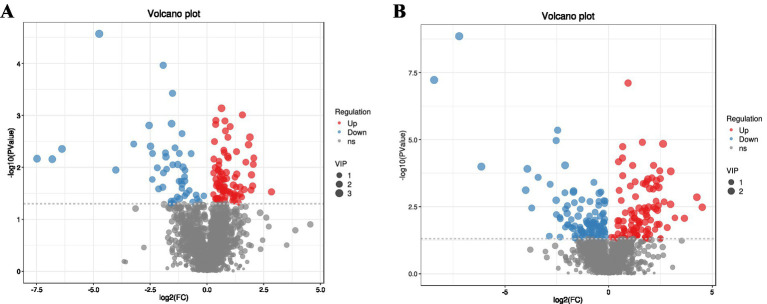
Combined differential volcano plot for positive and negative ion modes. **(A)** Positive ion mode. **(B)** Negative ion mode. The *x*-axis represents the log₂-transformed fold change (log₂FC) in metabolite expression between groups. The *y*-axis represents the -log₁₀(*p*-value), indicating the statistical significance of expression changes. Both axes are on a logarithmic scale. Each point represents a metabolite, with its size corresponding to the variable importance in projection (VIP) value. By default, red points denote significantly upregulated metabolites, blue points denote significantly downregulated metabolites, and gray points denote non-significant metabolites.

Figure 8 presents the screening strategy for differential serum metabolites, which applied the thresholds of variable importance in projection (VIP) > 1 and *p <* 0.05 within the orthogonal partial least squares-discriminant analysis (OPLS-DA) framework. Metabolites satisfying both criteria were classified as differential trend metabolites. Following identification, 345 metabolites were characterized as differential, including 186 up-regulated and 159 down-regulated species.

#### 3.4.7 KEGG pathway enrichment analysis

**Figure 9:**
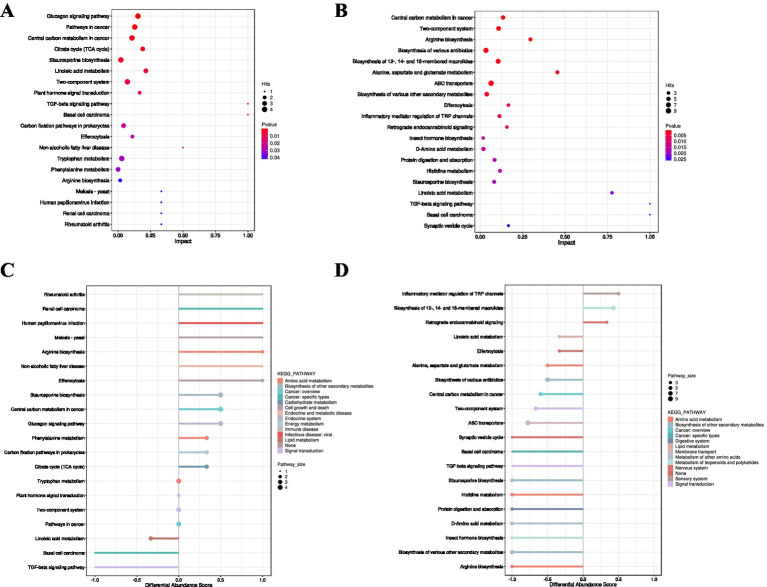
KEGG pathway analysis. **(A,B)** Enrichment analysis bubble plots (Control vs. Experimental Group I and II). **(C,D)** Differential abundance (DA) score plots (Control vs. Experimental Group I and II). **(A,B)** The *x*-axis represents the enrichment significance (−log₁₀(*p*-value)). The *y*-axis lists KEGG pathways. Bubble size indicates the number of differential metabolites enriched in the corresponding pathway. **(C,D)** The *x*-axis represents the differential abundance (DA) score, reflecting the overall expression trend of all annotated metabolites within a pathway (1 = entirely upregulated, −1 = entirely downregulated). The *y*-axis lists KEGG pathway names. Dot size indicates the count of annotated differential metabolites within the pathway. Segments to the right/left of the central axis indicate overall upregulation/downregulation trends, respectively, with length representing the absolute DA score.

Figure 9A illustrates that, among the top 20 enriched pathways differentiating the Control Group from Experimental Group I, nine metabolic pathways exhibited highly significant differences (*p* < 0.01). These included the Two-component system, Inflammatory mediator regulation of TRP channels, Efferocytosis, Central carbon metabolism in cancer, Biosynthesis of various other secondary metabolites, Arginine biosynthesis, Insect hormone biosynthesis, Staurosporine biosynthesis, and Alanine, aspartate and glutamate metabolism.

Figure 9B shows that, within the top 20 enriched pathways between the Control Group and Experimental Group II, nine metabolic pathways also displayed highly significant differences (*p* < 0.01). These comprised Central carbon metabolism in cancer, Two-component system, Arginine biosynthesis, Biosynthesis of various antibiotics, Biosynthesis of 12-, 14-, and 16-membered macrolides, Alanine, aspartate and glutamate metabolism, ABC transporters, Biosynthesis of various other secondary metabolites, and Efferocytosis.

The analysis suggests that the majority of differential metabolites were functionally associated with the biological processes governed by these nine pathways.

#### 3.4.8 Differential abundance of KEGG pathways

In Figure 9C, comparison between the Control Group and Experimental Group I revealed 13 metabolic pathways exhibiting an upward trend, including *Rheumatoid arthritis, Renal cell carcinoma, Human Papillomavirus infection, Meiosis-yeast, Arginine biosynthesis, Non-alcoholic fatty liver disease, Efferocytosis, Staurosporine biosynthesis, Central carbon metabolism in cancer, Glucagon signaling pathway, Phenylalanine metabolism, Carbon fixation pathways in prokaryotes*, and the *Citrate cycle*. Among them, Rheumatoid arthritis, Renal cell carcinoma, Human Papillomavirus infection, Meiosis-yeast, Arginine biosynthesis, Non-alcoholic fatty liver disease, and Efferocytosis displayed stronger up-regulation trends relative to the remaining pathways. In contrast, three pathways demonstrated a downward trend, namely *Linoleic acid metabolism, Basal cell carcinoma*, and *TGF-beta signaling pathway*.

In Figure 9D, comparison between the Control Group and Experimental Group II indicated three pathways with an up-regulated tendency, specifically *Inflammatory mediator regulation of TRP channels, Biosynthesis of 12-, 14*-, *and 16-membered macrolides, and Retrograde endocannabinoid signaling.* Seventeen pathways exhibited a down-regulated tendency, including *Linoleic acid metabolism, Efferocytosis, Alanine, aspartate and glutamate metabolism, Biosynthesis of* var*ious antibiotics, Central carbon metabolism in cancer, Two-component system* (commonly *referring to* bacterial *signal transduction*)*, ABC transporters, Synaptic vesicle cycle, Basal cell carcinoma, TGF-beta signaling pathway, Staurosporine biosynthesis, Histidine metabolism, Protein digestion and absorption, D-Amino acid metabolism, Biosynthesis of various other secondary metabolites, and Arginine biosynthesis.*

## 4 Correlation analysis

### 4.1 Correlation analysis between rumen microbiota and intestinal microbiota of Simmental cows after supplementary feeding of L-Citrulline

#### 4.1.1 Correlation analysis between rumen microbiota and intestinal microbiota at the phylum level

**Figure 10:**
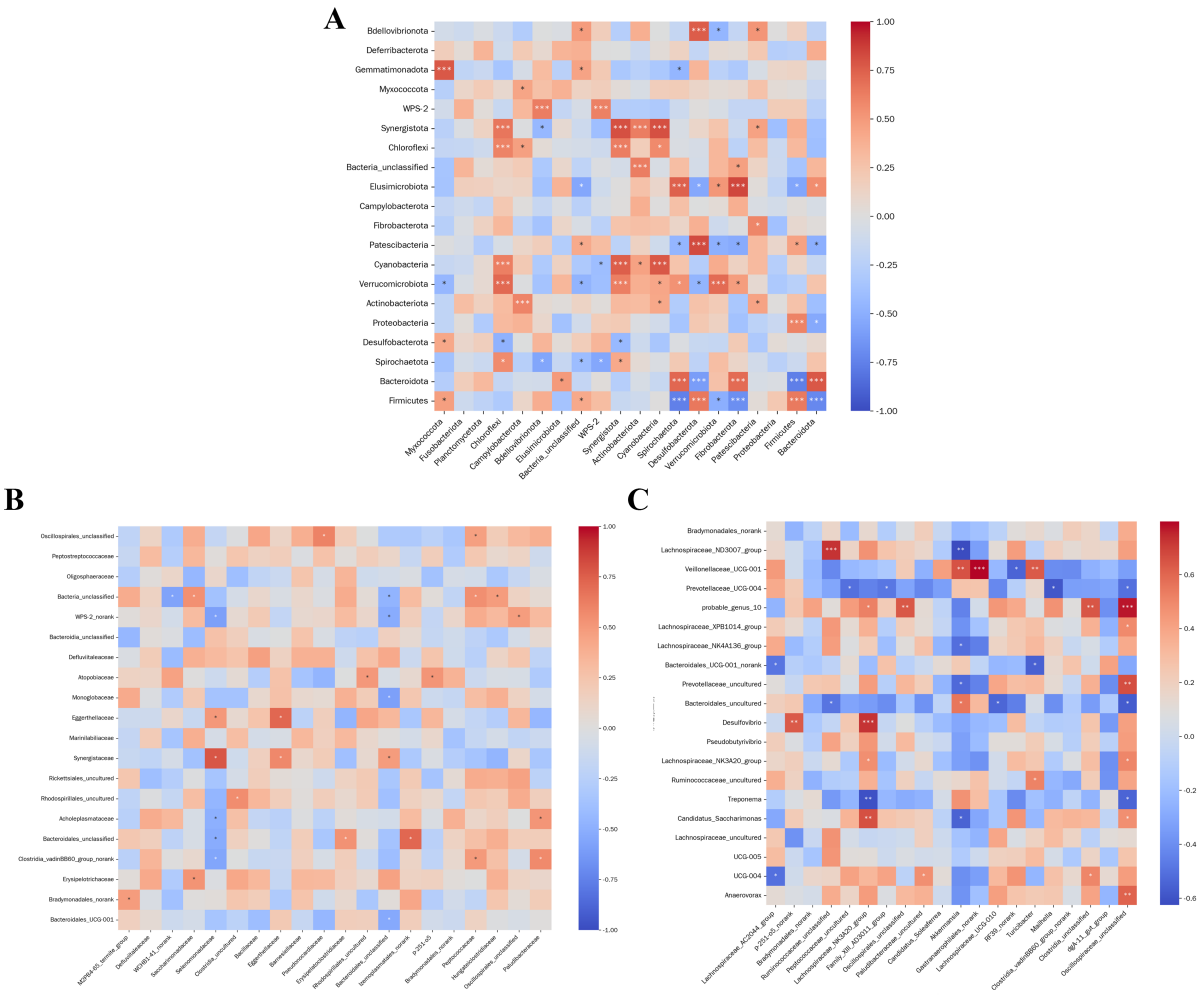
Correlation analysis between rumen and intestinal microbiota at different taxonomic levels. **(A)**. Phylum level. **(B)** Family level. **(C)** Genus level. The x-axis and *y*-axis represent intestinal and rumen microbiota, respectively. Cell colors correspond to correlation coefficients (*R*-values). Asterisks denote statistical significance: * 0.01 < *p* ≤ 0.05, ** 0.001 < *p* ≤ 0.01, *** *p* ≤ 0.001.

The correlation heatmap depicts associations between species and environmental variables, reflecting both the magnitude and significance of relationships across multiple environmental factors and microbial taxa. In Figure 10A, analysis of rumen and intestinal microbiota at the phylum level in Simmental cows revealed the following: *Bdellovibrionota* abundance displayed a highly significant correlation with WPS-2 abundance (*p <* 0.001); *Verrucomicrobiota* abundance was negatively correlated with *Firmicutes* (0.01 *< p ≤* 0.01); *Firmicutes* abundance exhibited a strong positive correlation with *Bacteroidota* (*p <* 0.001); and reciprocally, *Bacteroidota* abundance was also positively correlated with *Firmicutes* (*p <* 0.001).

#### 4.1.2 Correlation analysis between rumen microbiota and intestinal microbiota at the family level

As illustrated in Figure 10B, family-level analysis indicated that *Eggerthellaceae* abundance correlated positively with *Synergistaceae* (0.01 *< p ≤* 0.01). Unclassified Bacteria abundance showed a negative correlation with *WPS-2_norank* (0.01 *< p ≤* 0.01) while being positively correlated with *Synergistaceae*. *Selenomonadaceae* abundance was positively correlated with *Synergistaceae* (0.01 *< p ≤* 0.01) but negatively correlated with *Clostridia_vadinBB60_group_norank* (0.01 *< p ≤* 0.01). In addition, *Erysipelatoclostridiaceae* abundance demonstrated a significant positive correlation with unclassified *Bacteroidales* (0.01 *< p ≤* 0.01).

#### 4.1.3 Correlation analysis between rumen microbiota and intestinal microbiota at the genus level

As illustrated in Figure 10C, correlation analysis of ruminal and intestinal microbiota at the genus level in Simmental cows revealed several significant associations. The abundance of uncultured *Ruminococcaceae* exhibited a highly significant positive correlation with *Lachnospiraceae_ND3007_group* (*p <* 0.001) and a significant negative correlation with unclassified *Bacteroidales* (0.01 *< p ≤* 0.05). *Lachnospiraceae_NK3A20_group* abundance was strongly and positively correlated with *Desulfovibrio* (*p <* 0.001), while showing a negative association with *Treponema* (0.001 *< p ≤* 0.01). *Gastranaerophilales_norank* displayed a highly significant positive correlation with *Veillonellaceae_UCG-001* (*p <* 0.001). Furthermore, uncultured *Oscillospiraceae* abundance correlated positively with *probable_genus_10* (*p <* 0.001) and uncultured *Prevotellaceae* (0.001 *< p ≤* 0.01), but negatively with unclassified *Bacteroidales* (0.01 *< p ≤* 0.05).

### 4.2 Correlation analysis between intestinal microbiota (phylum level) and serum metabolites of Simmental cows after supplementary feeding of L-Citrulline

#### 4.2.1 Correlation analysis between intestinal microbiota (phylum level) and serum metabolites

**Figure 11:**
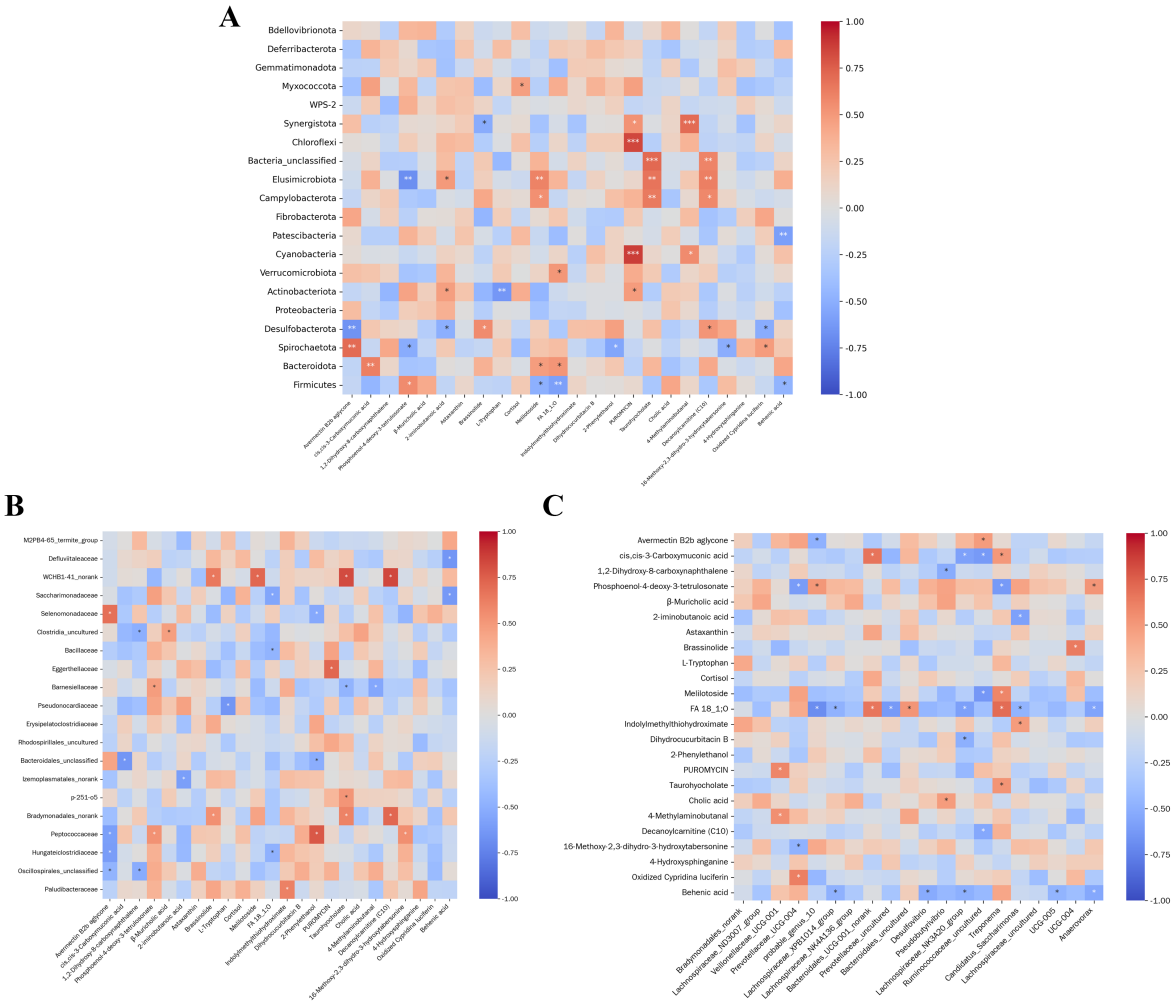
Correlation analysis between intestinal microbiota and serum metabolites at different taxonomic levels. **(A)** Phylum level. **(B)** Family level. **(C)** Genus level. The *x*-axis and *y*-axis *rep*resent serum metabolites and intestinal microbiota, respectively. Cell colors correspond to correlation coefficients (*R*-values). Asterisks denote statistical significance: *0.01 < *p* ≤ 0.05, **0.001 < *p* ≤ 0.01, ***p ≤ 0.001.

As shown in Figure 11A, several significant associations were identified. The abundance of 4-Methylcatechol exhibited a strongly positive correlation with *Synergistota* (*p <* 0.001). Taurohyocholate abundance displayed a strongly positive correlation with unclassified Bacteria (*p <* 0.001), a highly positive correlation with *Elusimicrobiota* (0.001 *< p ≤* 0.01), and a positive correlation with *Campylobacterota* (0.01 < p ≤ 0.05). PURPURIN abundance was strongly correlated with *Cyanobacteria* (*p <* 0.001). Avermectin B2b aglycone abundance showed a highly negative correlation with *Desulfobacterota* (0.001 *< p ≤* 0.01);

Behenic acid abundance was negatively correlated with *Patescibacteria* (0.001 *< p ≤* 0.01) and also showed a weaker negative correlation with *Firmicutes* (0.01 *< p ≤* 0.05). Cis,cis-3-Carboxymuconic acid abundance exhibited a highly positive correlation with *Bacteroidota* (0.001 *< p ≤* 0.01);

Decanoylcarnitine (C10) abundance demonstrated strong positive correlations with *unclassified* Bacteria (*p <* 0.001), *Elusimicrobiota* (0.001 *< p ≤* 0.01), and *Campylobacterota* (0.001 *< p ≤* 0.01).

#### 4.2.2 Correlation analysis between intestinal microbiota (family level) and serum metabolites

Figure 11B Demonstrates that specific metabolites exhibited significant correlations with microbial taxa. The abundance of 16-methoxy-2,3-dihydro-3-hydroxytabersonine *was p*ositively associated with *Peptococcaceae* (0.01 *< p ≤* 0.01), whereas 4-methylaminobutanal abundance showed a negative association with *Barnesiellaceae* (0.01 *< p ≤* 0.01). A positive association was also identified between 2-phenylethanol *abundance and Peptococcaceae* (0.01 *< p ≤* 0.01). In contrast, 2-iminobutanoic acid abundance correlated negatively with *Izimaserumtales_norank* (0.01 *< p ≤* 0.01). Additionally, behenic *acid abundance disp*layed negative associations with both *Defluviitaleaceae* and *Selenomonadaceae* (0.01 *< p ≤* 0.01).

#### 4.2.3 Correlation analysis between intestinal microbiota (genus level) and serum metabolites

As presented in Figure 11C, several associations were identified. *Veillonellaceae_UCG-001* abundance exhibited significant positive correlations with both 4-Methylaminobutanal (0.01 *< p ≤* 0.01) and PUROMYCIN (0.01 *< p ≤* 0.01). *UCG-005* abundance displayed a significant negative correlation with Behenic acid (0.01 *< p ≤* 0.01), while *Anaerovorax* abundance also showed a significant negative correlation with Behenic acid (0.01 *< p ≤* 0.01); *Treponema* abundance demonstrated significant positive correlations with Taurocholate (0.01 *< p ≤* 0.01) and FA 18_1,0 (0.01 *< p ≤* 0.01). *Ruminococcaceae_NK3A20_group* abundance correlated negatively with both cis,cis-3-Carboxymuconic acid (0.01 *< p ≤* 0.01) and FA 18_1,0 (0.01 *< p ≤* 0.01).

### 4.3 Correlation analysis between serum metabolites and serum reproductive hormones of Simmental cows after supplementary feeding of L-Citrulline

**Figure 12:**
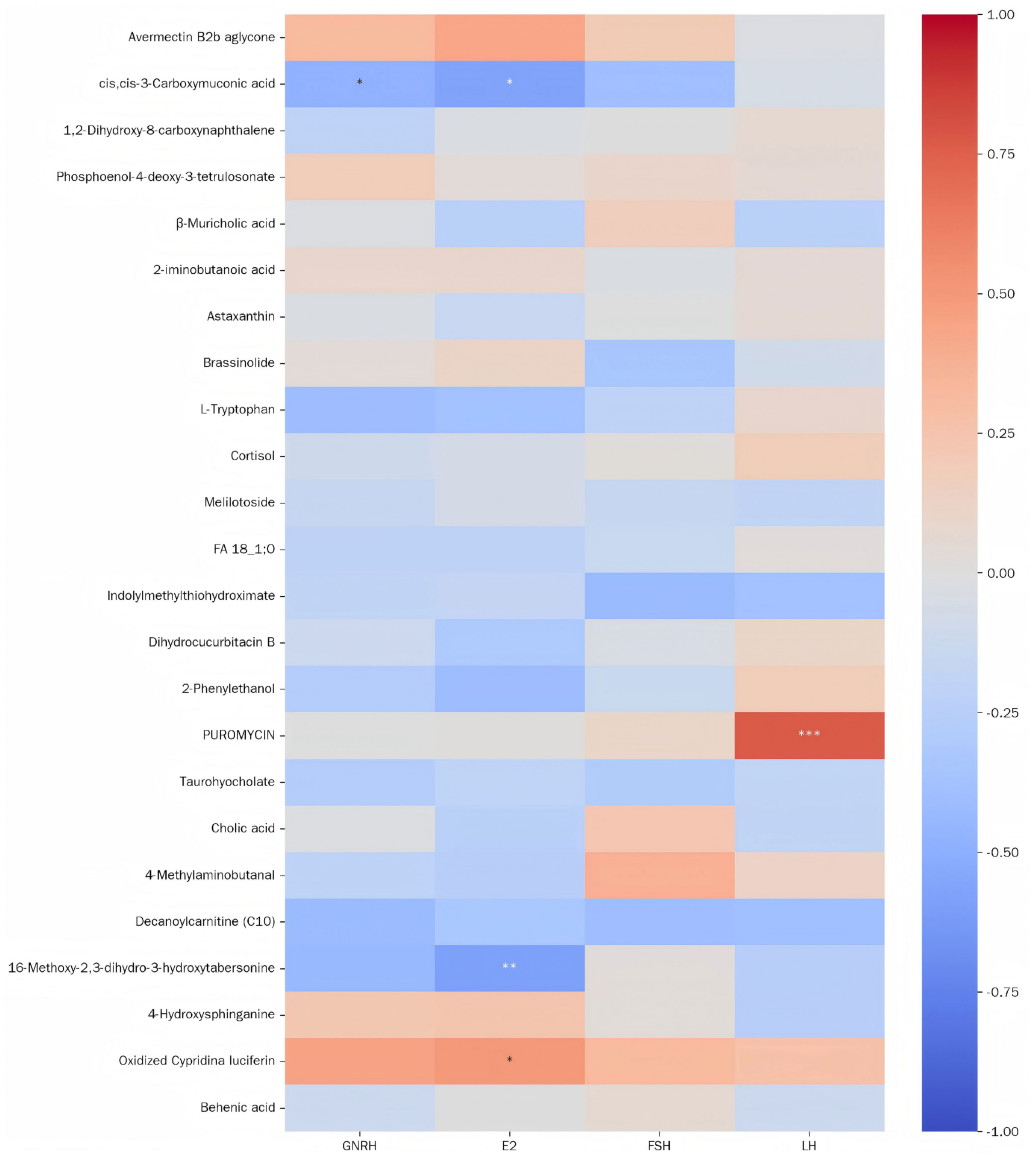
Heatmap of correlation analysis between serum differential metabolites and reproductive hormones. The *x*-axis and *y*-axis *rep*resent metabolite names and reproductive hormones, respectively. Cell color indicates the correlation coefficient between a metabolite and a hormone. Asterisks denote significance levels: *0.01 < *p* ≤ 0.05, **0.001 < *p* ≤ 0.01, *** p ≤ 0.001.

As illustrated in Figure 12, cis,cis-3-Carboxymuconic acid abundance showed significant negative correlations with GnRH and E_2_ (0.01 *< p ≤* 0.01). 16-Methoxy-2,3-dihydro-3-hydroxytabersonine abundance was strongly negatively correlated with E_2_ (0.01 *< p ≤* 0.01). Oxidized Cypridina luciferin abundance demonstrated a significant positive correlation with E_2_ (0.01 *< p ≤* 0.01). Puromycin abundance exhibited a highly significant positive correlation with LH (*p* < 0.001).

## 5 Discussion

The rumen and intestinal microbial ecosystems of ruminants are interconnected through microbial translocation, metabolic exchange, and host physiological modulation (Li et al., 2022). Distinct taxonomic structures and metabolic functions characterize the two communities, yet their coordinated activity enables comprehensive and efficient utilization of ingested nutrients (Jami et al., 2013). Stability of this system relies on intrinsic dynamic regulation of abundance and composition (Martínez-Fernández et al., 2015). The rumen is predominantly occupied by *Firmicutes* and *Bacteroidota* (>80% of total), enriched with cellulolytic taxa such as *Ruminococcus* and Fibrobacter, together with methanogens including *Methanobrevibacter*, which jointly drive the cascade of fiber degradation and methane formation (Belanche et al., 2012; Snelling et al., 2019). In contrast, the intestinal microbiota is characterized by *Proteobacteria* and *Actinobacteriota*, with enrichment of mucin-degrading genera such as *Akkermansia* and butyrate-producing bacteria such as *Roseburia*, functioning mainly in secondary fermentation of host-derived carbohydrates. These communities interact in hydrogen and nitrogen turnover: H₂ generated during ruminal fermentation is transferred to the intestine, where it serves as a substrate for acetate-producing bacteria such as *Syntrophomonas* and methanogens such as *Methanosphaera*, thereby alleviating hydrogen-associated inhibition of ruminal fiber breakdown (Lu et al., 2024; Zhang et al., 2025). Microbial protein synthesized within the rumen undergoes digestion in the intestine, and the ammonia released is recycled by the rumen, forming a continuous “rumen–intestinal nitrogen cycle” (Reynolds and Kristensen, 2008).

In Experimental Group I, supplementation with *L-*Cit significantly increased the relative abundance of rumen *Bacteroidota* compared with the control group. Members of *Bacteroidota* produce cellulase and hemicellulase, thereby enhancing crude fiber degradation and promoting the generation of rumen acetate and propionate, which supply essential energy during the estrus period (Yin et al., 2024). In the *L-*Cit-supplemented groups, *Prevotellaceae* abundance reached 20.38%, with the argininosuccinate lyase gene (argG) encoded in its genome supporting the conversion of *L-*Cit to arginine (Chacher et al., 2012; Si et al., 2021).

Following *L-*Cit supplementation, Experimental Group II exhibited a marked increase in the relative abundance of intestinal *Firmicutes*. Members of this phylum produce polysaccharide-degrading enzymes that enhance the breakdown of carbohydrates, including crude fiber residues, thereby increasing the generation of short-chain fatty acid (SCFA) precursors such as pyruvate and lactic acid. These metabolites not only serve as energy sources for intestinal epithelial cells (Matheus et al., 2023) but also contribute to the reduction of harmful metabolite accumulation in the gut. This observation aligns with the conclusion of De Vadder et al. (2014), which proposed that SCFAs improve systemic metabolism through modulation of the gut–brain neural circuit and regulation of oxidative stress.

No significant intergroup variation in Alpha or Beta diversity was detected, suggesting that L-Cit, as a rumen-undegradable amino acid precursor, does not alter the fundamental ecological stability of ruminal and intestinal microbiota. Instead, the modulation remains confined to specific functional groups, thereby avoiding the microbiota disturbances often associated with high-dose additives (Zhao et al., 2024).

At the phylum level, rumen *Bacteroidota* exhibited a highly significant positive correlation with intestinal *Firmicutes*. Rumen *Bacteroidota* decomposes dietary crude fiber into VFAs such as acetate and propionate, while intestinal *Firmicutes* subsequently convert residual VFAs into butyrate. Circulating butyrate reaches the liver and contributes to gluconeogenesis, thereby supplying energy for follicular development (Matheus et al., 2023; Yang et al., 2024; Bazer et al., 2009; Turnbaugh et al., 2009). Greater VFA output from crude fiber degradation by rumen *Bacteroidota* corresponds to higher VFA conversion efficiency by intestinal *Firmicutes*, forming a self-sustaining energy loop. In contrast, rumen *Verrucomicrobiota* displayed a highly significant negative correlation with intestinal *Bacteroidota* and *Firmicutes*. The main substrate of rumen *Verrucomicrobiota* is mucopolysaccharide, for which degradation efficiency remains limited. Elevated *Verrucomicrobiota* abundance intensifies competition with intestinal *Bacteroidota* for the epithelial mucus layer, reducing *Bacteroidota* abundance and impairing crude fiber degradation (Kong et al., 2025). Increased *Verrucomicrobiota* also suppresses *Firmicutes* proliferation: diminished butyrate synthesis weakens the intestinal barrier, while reduced VFA utilization efficiency in the intestine results in energy loss (Pryde et al., 2002). Collectively, these processes account for the high *Verrucomicrobiota* abundance, low estrus rate, and decreased hormone levels observed in the control group.

At the family level, a highly significant positive association was identified between rumen *Synergistaceae* and intestinal *Eggerthellaceae* (0.001 *< p ≤* 0.01). *Synergistaceae* generate ammonia via dietary protein degradation, whereas *Eggerthellaceae* convert intestinal urea into ammonia, subsequently utilized for amino acid synthesis (Bach et al., 2019).

At the genus level, a highly significant positive association was detected between rumen *Veillonellaceae_UCG-001* and intestinal *Alkaliphila* (0.001 *< p ≤* 0.01), with both taxa enriched in Experimental Group I. *Veillonellaceae_UCG-001* convert lactic acid in the rumen to propionate, thereby preventing pH decline resulting from lactic acid accumulation. Stable rumen pH is essential for *Prevotellaceae*-mediated conversion of *L-*Cit to arginine (Wang et al., 2022). Intestinal *Alkaliphila* maintain pH homeostasis by secreting alkaline substances, creating a favorable environment for the hypothalamic–pituitary-ovarian (HPO) axis. This functional role aligns with the observed activation of the arginine biosynthesis pathway and elevated FSH levels in the experiment.

Nitric oxide (NO) exerts regulatory effects on the hypothalamic–pituitary-gonadal axis by modulating pulsatile GnRH secretion and controlling the release of FSH and LH, ultimately influencing ovarian steroidogenesis (Gobbetti and Zerani, 1998; Barnes et al., 2001). *L-*Cit supplementation markedly increased serum GnRH and FSH levels in Simmental cows (*p <* 0.05), consistent with findings reported in rams by Zhao et al. (2023). The elevation of GnRH may arise from the dual action of NO: attenuation of estradiol (E2)-mediated negative feedback on GnRH neurons, which alleviates inhibitory signaling, and direct activation of neuronal excitability and pulsatile secretion, thereby enhancing excitatory output (Goodman et al., 2022). The notable increase in estrus rate observed in *L-*Cit-treated groups suggests that *L-*Cit enhances follicular development and ovulation by improving the signaling efficiency of the HPO axis (Fan et al., 2024).

The PLS-DA model of serum untargeted metabolomics indicated a significant enrichment of the arginine metabolism pathway in Experimental Group I, suggesting a close association with nitrogen metabolism and immune regulation (Li et al., 2018). In Experimental Group II, however, arginine metabolism exhibited an initial activation followed by down-regulation, likely resulting from excessive arginine inhibiting ASS and ASL via negative feedback. Concurrently, surplus *L-*Cit may suppress intestinal arginine transporters, producing a discrepancy between marked pathway enrichment and insufficient metabolite availability, thereby failing to sustain hormone secretion. The TCA cycle pathway was markedly up-regulated in Experimental Group I, accompanied by elevated concentrations of citric acid, *α*-ketoglutarate, and succinate. Enhanced oxidative phosphorylation under these conditions may promote ATP production to support the pulsatile release of GnRH neurons in the hypothalamus (Zhang et al., 2021). Additionally, α-ketoglutarate, serving as a substrate for epigenetic modification, activated the expression of the follicle-stimulating hormone receptor (FSHR) gene in the ovary, consistent with the observed increase in FSH levels in this group.

KEGG analysis indicated that *L-*Cit indirectly enhanced energy and amino acid metabolism through activation of ruminal microbiota responsible for crude fiber degradation (Gilbreath et al., 2020). This shift was accompanied by down-regulation of linoleic acid metabolism, thereby reducing the efficiency of *γ*-linolenic acid conversion. The biphasic alteration of the arginine metabolism pathway, with initial up-regulation followed by down-regulation, implies a multifaceted role in host–microbe interactions that warrants further examination. Correlation analysis between metabolites and reproductive hormones revealed a significant positive association between Oxidized Cypridina luciferin and E_2_. Given its strong antioxidant capacity, increased abundance of Oxidized Cypridina luciferin indicates improved systemic oxidative defense. In Experimental Group I, the intestinal genus *Marvinbryantia* was enriched, a taxon known to secrete glutathione reductase, which enhances glutathione (GSH) regeneration and indirectly strengthens the antioxidant effect of Oxidized Cypridina luciferin (Liu and Fan, 2025). Excessive reactive oxygen species (ROS) can induce DNA damage and disrupt E_2_ synthesis, whereas Oxidized Cypridina luciferin supports E_2_ production by neutralizing ROS (Gilbreath et al., 2020).

**Figure 13:**
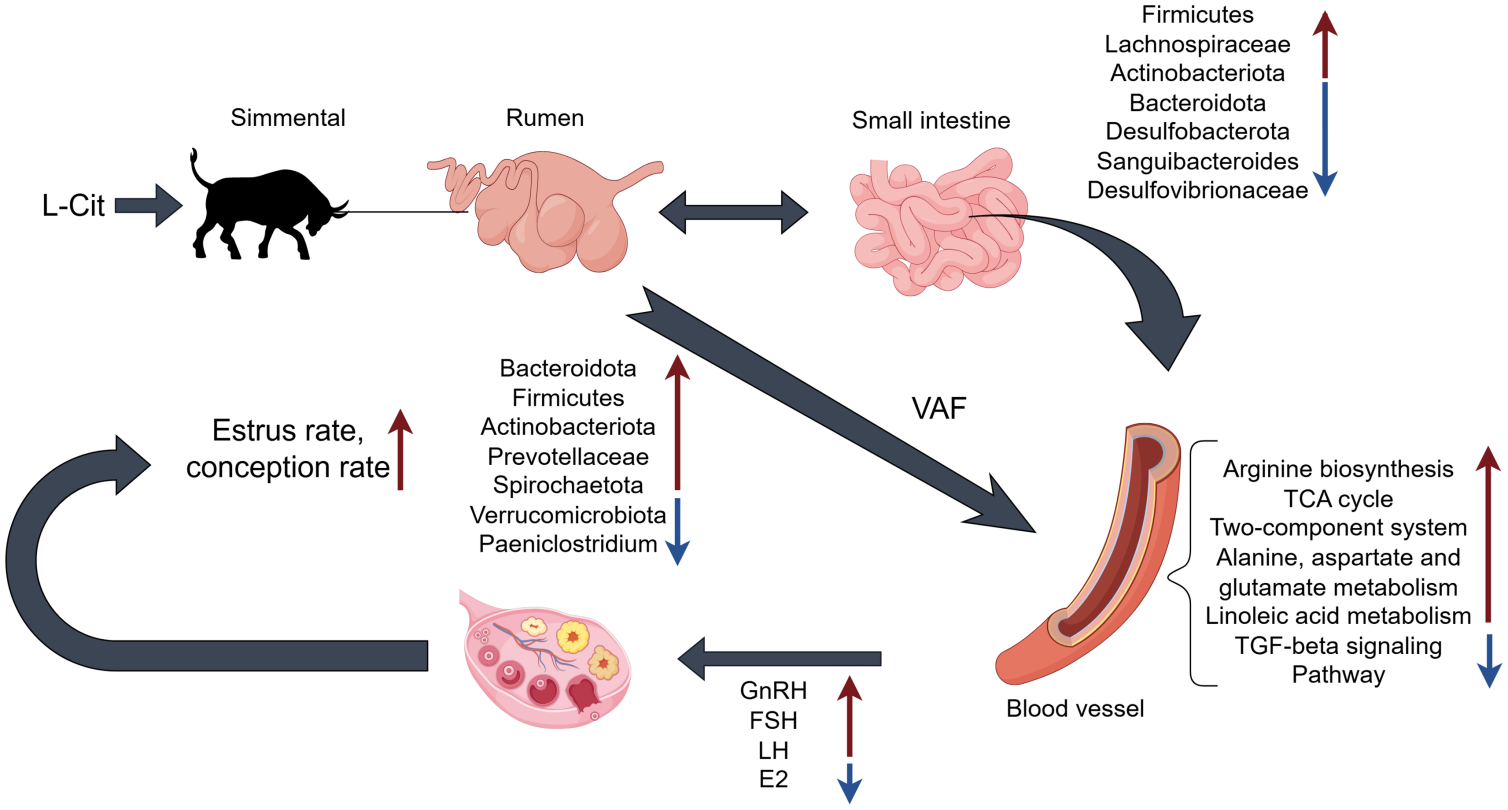
An integrative schematic overview of the effects of L-Citrulline supplementation on rumen microbiota, intestinal microbiota, serum metabolites, and reproductive hormones in Simmental cows.

Cit, as an endogenous precursor of arginine, serves as a key substrate in nitric oxide synthesis and reproductive regulation. Following absorption in the small intestine, nearly 80% of *L*-Cit undergoes conversion to arginine (Arg) in the kidneys, a process that preserves systemic Arg balance and modulates uterine-ovarian blood flow through NO-mediated vasodilation, thereby indirectly influencing the hypothalamic–pituitary-gonadal (HPG) axis (Morris 2002; Kone, 2004). Upon entering the duodenum, *L-*Cit is absorbed and subsequently transformed into Arg, which further contributes to systemic hemodynamics and the regulation of reproductive hormones, including GnRH, FSH, LH, and E_2_. Microbial taxa such as *Bacteroidota*, *Firmicutes*, *Actinobacteriota*, and the family *Prevotellaceae* constitute a complex ecological network that governs amino acid metabolism, encompassing Arg and its precursors. Functional pathways such as arginine biosynthesis, the *TCA cycle*, and *alanine-aspartate–glutamate* metabolism reveal microbial participation in host nitrogen metabolism (Mori et al., 2015). Within this framework, microorganisms provide additional *α*-ketoglutarate to ovarian granulosa cells, thereby enhancing E2 synthesis. The resulting modulation of blood metabolites and enrichment of associated metabolic pathways ultimately enhances reproductive efficiency in female animals (Figure 13).

## 6 Conclusion

Within the experimental framework, *L-*Cit supplementation modulated the relative abundance of microbial groups in the rumen and intestines, notably Bacteroidetes at the phylum level and *Prevotellaceae* at the family level. These microbial shifts were accompanied by alterations in serum metabolic pathways, including Arginine biosynthesis and the Citrate (TCA) cycle, which subsequently influenced serum hormone dynamics involving GnRH, FSH, LH, and E2. The integrated regulatory effects ultimately enhanced estrus expression and conception rates in cows. This study focused on Simmental cows in Xinjiang; thus, the results may not generalize to other breeds. Future work should examine endometrial receptivity and placental histology to elucidate implantation mechanisms.

## Data Availability

The data that supports the findings of this study are available from CNCB (https://ngdc.cncb.ac.cn), accession number PRJCA046881. Other data that supports the findings of this study are available from the corresponding author upon reasonable request.
