# Supplementary material for: Effects of L-citrulline supplementation in the basal diet on reproductive performance, serum metabolites, and microbial community structure in Simmental cows
Source: Front Microbiol. 2026 Mar 3;17:1742321. doi: 10.3389/fmicb.2026.1742321 (PMC12992054; doi:10.3389/fmicb.2026.1742321)
Supplement: Supplementary file 1 [file Data_Sheet_1.docx]

**Data Filtering and Processing Methods and Parameters**
The raw sequencing reads were processed according to the following quality control and filtering steps:

**Quality Trimming**:

The trailing bases of reads with a Phred quality score below 20 were trimmed.

A sliding window of 10 bp was applied: if the average quality score within the window dropped below 20, the read was truncated from the start of that window.

Reads shorter than 50 bp after quality control were discarded.

**Read Pair Merging:**

Paired-end reads were merged into single contigs based on their overlap region using the FLASH tool.

The minimum allowed overlap length was 10 bp.

The maximum mismatch ratio allowed in the overlap region was 0.2, and reads failing to meet these criteria were excluded.

**Sample Demultiplexing and Orientation Correction:**

Sequences were assigned to samples based on their unique barcode and primer sequences.

No mismatches were allowed in barcode recognition, while up to 2 mismatches were permitted in primer matching.

Sequence orientation was adjusted accordingly.

**Chimera Removal:**

Chimeric sequences were identified and filtered using the USEARCH software in combination with the GOLD database.

Both de novo and reference-based chimera detection strategies were applied.

The following software tools were utilized in the pipeline: Trimmomatic, FLASH, Usearch, QIIME, and custom Perl scripts.

**Taxonomic Assignment Reference Databases**

To obtain the taxonomic classification for each OTU, the representative sequences of OTUs clustered at 97% similarity were analyzed using the RDP Classifier Bayesian algorithm. Taxonomic composition was statistically summarized at each classification level: domain, kingdom, phylum, class, order, family, genus, and species.

The following reference databases were employed for alignment:

For 16S rRNA genes of Bacteria and Archaea (the SILVA database was used as the default unless otherwise specified):

SILVA (Release 132, http://www.arb-silva.de)

RDP (Release 11.5, http://rdp.cme.msu.edu/)

Greengenes (Release 13.8, http://greengenes.secondgenome.com/)

For fungal ITS regions:

UNITE (Release 7.1, http://unite.ut.ee/index.php) fungal database

For functional genes:

FGR (FunGene database, Release 7.3, http://fungene.cme.msu.edu/), a functional gene database curated by RDP from GenBank

Software and Algorithms:

Taxonomic assignment was performed using the QIIME pipeline (v1.9.0, http://qiime.org/scripts/assign_taxonomy.html) in combination with the RDP Classifier (version 2.2, http://sourceforge.net/projects/rdp-classifier/). A confidence threshold of 0.7 was applied for taxonomic assignments.

**Formulas for Alpha Diversity Indices**

**Chao1 Index Estimation**
The Chao1 index, initially developed by Chao (1984), is a nonparametric estimator commonly employed in ecology to approximate the total species richness within a community. In this study, it was utilized to estimate the number of operational taxonomic units (OTUs) present in each sample. The calculation was performed using the following formula:

Schao1​=Sobs​+2(n2​+1)/n1​(n1​−1)​

where:

Schao1​ = estimated total number of OTUs;

Sobs​ = number of OTUs actually observed;

​n1= number of OTUs containing only one sequence (i.e., "singletons");

n2 = number of OTUs containing only two sequences (i.e., "doubletons").

**The Simpson Index**

The Simpson index, proposed by Edward Hugh Simpson (1949), is one of the metrics used to estimate microbial diversity within a sample. It is commonly applied in ecology to quantitatively describe the biodiversity of a region. A higher Simpson index value indicates lower community diversity.

Dsimpson​=N(N−1)/∑i=1Sobs​​ni​(ni​−1)​

where:Sobs= number of observed OTUs;
ni= number of sequences in the *i*-th OTU;
N = total number of sequences.

**Shannon Index**

The Shannon index is a metric used to estimate microbial diversity within a sample. Along with the Simpson diversity index, it is commonly applied to reflect alpha diversity. A higher Shannon index value indicates greater community diversity.

Hshannon​=−∑i=1Sobs​​N/ni​​lnN/ni​​

where:

Sobs= the number of observed OTUs;
ni= the number of sequences contained in the *i*-th OTU;
N = the total number of sequences.

**Coverage**

Coverage refers to the sequencing depth of each sample library. A higher value indicates a greater probability that sequences in the sample have been detected, and a lower probability that they remain undetected. This index reflects whether the current sequencing results represent the true microbial composition of the sample.

C=1−N/na​​

where:
ni= the number of sequences contained in the *i*-th OTU;
N = the total number of sequences.

**Analysis Software:**
Alpha diversity indices were calculated using mothur (version v.1.41.0) following the standard operating procedure outlined by Schloss et al. (available at: <http://www.mothur.org/wiki/Schloss_SOP#Alpha_diversity>). All indices were evaluated based on operational taxonomic units (OTUs) clustered at a 97% similarity threshold (0.97).

**Beta Diversity Intergroup Distance Analysis**

The degree of dissimilarity in species or functional abundance distributions between samples was quantified using statistical distance metrics. The Bray-Curtis distance was calculated for each pair of samples to generate a distance matrix, which was subsequently used for downstream beta diversity analysis and visual statistical interpretation. Visualization of this distance matrix via a heatmap provided an intuitive overview of the distribution of dissimilarities among samples.

To compare the dispersion of distances within and between different sample groups (e.g., based on classification or environmental factors), the distance matrix was subjected to interquartile range calculation for each group. Differences in the distribution of distances among groups were statistically evaluated using multiple two-sample Student's t-tests to determine significant intergroup distinctions.

**Differential Abundance Analysis**

Differentially abundant features were first identified using the non-parametric factorial Kruskal–Wallis (KW) sum-rank test, which detects taxa with significant abundance differences across groups. Subsequently, LEfSe (Linear Discriminant Analysis Effect Size) was applied to estimate the effect size of each taxon’s contribution to the observed differences using linear discriminant analysis (LDA).

Analysis Software

LEfSe (available at http://huttenhower.sph.harvard.edu/galaxy/root?tool_id=lefse_upload) was employed to perform linear discriminant analysis (LDA) based on taxonomic composition, comparing samples under different grouping conditions. This method identifies microbial taxa or community features that significantly drive separation between sample groups.

**LC-MS/MS Analysis of Serum Metabolites**

Reagents and Materials

Acetonitrile (Fisher); Methanol (Fisher); Ultrapure water (Fisher); LC vials (Agilent); Chromatographic column: 1.7 μm, 2.1 × 100 mm (Waters).

Sample Preparation

Eighteen Simmental cow serum samples were thawed on ice after removal from a −80°C freezer. A 100 μL aliquot of each serum sample was transferred to a 1.5 mL microcentrifuge tube, followed by the addition of 400 μL of a methanol:acetonitrile (1:1, v/v) mixture. The mixture was vortexed vigorously for 30 s and then subjected to ultrasonication for 10 min. After sonication, samples were kept at −20°C for 1 h, followed by centrifugation at 13,000 rpm for 15 min at 4°C. A 300 μL portion of the supernatant was transferred and lyophilized in a vacuum freeze-dryer. The dried residue was reconstituted in an acetonitrile:water (1:1, v/v) solution, vortexed for 30 s, and sonicated for 10 min. After a second centrifugation under the same conditions (13,000 rpm, 15 min, 4°C), 50 μL of the supernatant was transferred to an LC vial for LC-MS analysis.

Sample Analysis

Liquid Chromatography Conditions

Column: Waters ACQUITY UPLC BEH Amide (1.7 µm, 2.1 mm × 100 mm);

Mobile phase: A) ultrapure water containing 25 mM ammonium acetate and 25 mM ammonia, B) acetonitrile;

Flow rate: 0.5 mL/min; Column temperature: 40°C; Injection volume: 2 μL.

The LC gradient program is shown in Table 1.

Mass Spectrometry Conditions

Ion source temperature: 650°C; Ion spray voltage: +5,500 V (positive ion mode) / −4,500 V (negative ion mode); Declustering potential: 60 V;Ion source gas: Gas 1: 60 psi, Gas 2: 60 psi; Curtain gas: 30 psi; Collision-induced dissociation parameter was set to high.

**Table1 Liquid phase elution gradient**

| **Time** | **A（%）** | **B（%）** | Flow Rate（mL/min） |
| --- | --- | --- | --- |
| 0 | 5 | 95 | 0.5 |
| 0.5 | 5 | 95 | 0.5 |
| 7 | 35 | 65 | 0.5 |
| 8 | 60 | 40 | 0.5 |
| 9 | 60 | 40 | 0.5 |
| 9.1 | 5 | 95 | 0.5 |
| 12 | 5 | 95 | 0.5 |
